# Assessment of a Diagnostic Classification System for Management of Lesions to Exclude Melanoma

**DOI:** 10.1001/jamanetworkopen.2021.34614

**Published:** 2021-12-10

**Authors:** Ian Katz, Blake O’Brien, Simon Clark, Curtis T. Thompson, Brian Schapiro, Anthony Azzi, Alister Lilleyman, Terry Boyle, Lore Jane L. Espartero, Miko Yamada, Tarl W. Prow

**Affiliations:** 1 Southern Sun Pathology, Sydney, New South Wales, Australia; 2 University of Queensland, Brisbane, Queensland, Australia; 3 Sullivan Nicolaides Pathology, Brisbane, Queensland, Australia; 4 Douglass Hanly Moir Pathology, Sydney, New South Wales, Australia; 5 CTA Pathology, Portland, Oregon; 6 Newcastle Skin Check, Charlestown, New South Wales, Australia; 7 Allied Health and Human Performance, University of South Australia, Adelaide, South Australia, Australia; 8 Future Industries Institute, University of South Australia, Adelaide, South Australia, Australia

## Abstract

**Importance:**

The proposed MOLEM (Management of Lesion to Exclude Melanoma) schema is more clinically relevant than Melanocytic Pathology Assessment Tool and Hierarchy for Diagnosis (MATH-Dx) for the management classification of melanocytic and nonmelanocytic lesions excised to exclude melanoma. A more standardized way of establishing diagnostic criteria will be crucial in the training of artificial intelligence (AI) algorithms.

**Objective:**

To examine pathologists’ variability, reliability, and confidence in reporting melanocytic and nonmelanocytic lesions excised to exclude melanoma using the MOLEM schema in a population of higher-risk patients.

**Design, Setting, and Participants:**

This cohort study enrolled higher-risk patients referred to a primary care skin clinic in New South Wales, Australia, between April 2019 and December 2019. Baseline demographic characteristics including age, sex, and related clinical details (eg, history of melanoma) were collected. Patients with lesions suspicious for melanoma assessed by a primary care physician underwent clinical evaluation, dermoscopy imaging, and subsequent excision biopsy of the suspected lesion(s). A total of 217 lesions removed and prepared by conventional histologic method and stained with hematoxylin-eosin were reviewed by up to 9 independent pathologists for diagnosis using the MOLEM reporting schema. Pathologists evaluating for MOLEM schema were masked to the original histopathologic diagnosis.

**Main Outcomes and Measures:**

Characteristics of the lesions were described and the concordance of cases per MOLEM class was assessed. Interrater agreement and the agreement between pathologists’ ratings and the majority MOLEM diagnosis were calculated by Gwet AC1 with quadratic weighting applied. The diagnostic confidence of pathologists was then assessed.

**Results:**

A total of 197 patients were included in the study (102 [51.8%] male; 95 [48.2%] female); mean (SD) age was 64.2 (15.8) years (range, 24-93 years). Overall, 217 index lesions were assessed with a total of 1516 histological diagnoses. Of 1516 diagnoses, 677 (44.7%) were classified as MOLEM class I; 120 (7.9%) as MOLEM class II; 564 (37.2%) as MOLEM class III; 114 (7.5%) as MOLEM class IV; and 55 (3.6%) as MOLEM class V. Concordance rates per MOLEM class were 88.6% (class I), 50.8% (class II), 76.2% (class III), 77.2% (class IV), and 74.2% (class V). The quadratic weighted interrater agreement was 91.3%, with a Gwet AC1 coefficient of 0.76 (95% CI, 0.72-0.81). The quadratic weighted agreement between pathologists’ ratings and majority MOLEM was 94.7%, with a Gwet AC1 coefficient of 0.86 (95% CI, 0.84-0.88). The confidence in diagnosis data showed a relatively high level of confidence (between 1.0 and 1.5) when diagnosing classes I (mean [SD], 1.3 [0.3]), IV (1.3 [0.3]) and V (1.1 [0.1]); while classes II (1.8 [0.2]) and III (1.5 [0.4]) were diagnosed with a lower level of pathologist confidence (≥1.5). The quadratic weighted interrater confidence rating agreement was 95.2%, with a Gwet AC1 coefficient of 0.92 (95% CI, 0.90-0.94) for the 1314 confidence ratings collected. The confidence agreement for each MOLEM class was 95.0% (class I), 93.5% (class II), 95.3% (class III), 96.5% (class IV), and 97.5% (class V).

**Conclusions and Relevance:**

The proposed MOLEM schema better reflects clinical practice than the MPATH-Dx schema in lesions excised to exclude melanoma by combining diagnoses with similar prognostic outcomes for melanocytic and nonmelanocytic lesions into standardized classification categories. Pathologists’ level of confidence appeared to follow the MOLEM schema diagnostic concordance trend, ie, atypical naevi and melanoma in situ diagnoses were the least agreed upon and the most challenging for pathologists to confidently diagnose.

## Key Points

**Question:**

What is the reliability of a more clinically focused, modified diagnostic classification system for diagnosing all lesions (ie, melanocytic and nonmelanocytic) excised to exclude melanoma and how is it associated with pathologists’ diagnostic confidence?

**Findings:**

In this cohort study that included 197 patients with suspected melanoma, the interrater agreement overall of Management of Lesion to Exclude Melanoma (MOLEM) diagnosis vs majority diagnosis and by mean confidence level of pathologists was excellent. The overall accuracy of pathologist compared with the majority diagnosis based on concordance rates per MOLEM class was 88.6% (class I), 50.8% (class II), 76.2% (class III), 77.2% (class IV), and 74.5% (class V).

**Meaning:**

These results suggest that combining melanocytic and nonmelanocytic lesions excised to exclude melanoma with similar prognostic outcomes into standardized classification categories may be more clinically relevant than the previous schema.

## Teaser

This cohort study examines pathologist assessments of skin lesions with a high risk for melanoma presented at an Australian primary care skin clinic using a novel classification schema.

## Introduction

The criterion standard diagnosis for melanoma is morphological assessment of hematoxylin-eosin–stained sections of biopsied material. However, there may be discordance between pathologists in the histologic distinction of melanoma from benign lesions in a significant number of so-called borderline lesions.^1,2,3^ For example, accuracy versus an expert panel has been reported to be as low as 45% for lesions including moderately dysplastic naevi and melanoma in situ.^3^ This level of discordance is likely to be because of the complexity of morphology within these melanocytic lesions combined with subjective diagnostic criteria,^4^ with difficult lesions showing overlapping histopathological features of benign naevus and melanoma. The misdiagnosis of melanoma has consequences for both patients and pathologists. In the US, the most common malpractice claim in dermatopathology practice is for the misdiagnosis of melanoma.^5^ In contrast, overdiagnosis of melanoma in ultimately benign lesions may lead to unnecessary surgeries. Titus et al^5^ reported that 1 in 4 US pathologists upgrade melanocytic skin lesions due to malpractice concerns, which could contribute to discordant diagnoses and lead to unnecessary care.

The American College of Radiology established a standard (BI-RADS [Breast Imaging-Reporting and Data System]) to address a similar lack of international standardization in radiology, which has since been adapted to melanocytic skin lesion pathology via the Melanocytic Pathology Assessment Tool and Hierarchy for Diagnosis (MPATH-Dx) reporting schema for melanocytic proliferations.^6^ Moving from a natural language diagnostic reporting scheme to a numerical system could have substantial benefits for quality of care and economy of service. Further, a numerical system could be a more robust bridge to computer-aided diagnosis and patient management.

Artificial intelligence (AI), including machine learning, has the potential to be useful in the interpretation of medical images. The algorithms used to train machines use a process called supervised learning. Having an accurate standard truth is vital in this process. It is evident that without a definite standard, the training may not be accurate.^7^ It is postulated that a diagnosis made by machine learning algorithms, especially of early-stage melanoma, may be more consistent and more replicable than human interpretation, but not necessarily close to the truth.^7^ A numerical classification scheme is attractive as a way of training an AI algorithm. However, the weakness of such method is that an algorithm cannot weight any data and will learn equally from any 2 cases in the same diagnostic class, irrespective of how challenging it was for human pathologists to classify the lesion.

When physicians become overconfident, there may be a miscalibration or misalignment between diagnostic accuracy and confidence. As a result, higher confidence may contribute to diagnostic errors.^8^ Although the original MPATH-Dx study suggested that an estimate of diagnostic confidence be added to a report of a melanocytic lesion,^5^ there are no subsequent publications to support this finding.

In practice, when pathologists are diagnosing melanoma, there are many types of confounding lesions that are nonmelanocytic in origin. Some of these include pigmented actinic keratosis, large cell acanthoma, seborrheic keratosis, and lichen planus such as keratosis, cysts, dermatofibroma, basal cell carcinoma, and squamous cell carcinoma.^9^ To our knowledge, there were no publications that examine the diagnostic variability in this entire spectrum of lesions excised to exclude melanoma at the time of writing. In this current study, the previously reported MPATH-Dx schema^6^ was extended into a 5-class scheme, which we have termed Management of Lesions to Exclude Melanoma (MOLEM), to include melanocytic and nonmelanocytic lesions in the categories.^10^

In this study, pathologists have used the MOLEM reporting scheme and included a measure of diagnostic confidence. It is hypothesized that, in the study population, compared with the MPATH-Dx, there would be similar variability in diagnosing so-called borderline lesions (ie, class II in the proposed MOLEM schema) and that this would be associated with confidence in diagnosis.

## Methods

### Study Design

**Figure 1.**
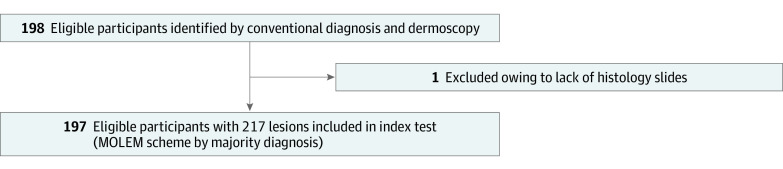
Participant Flowchart MOLEM indicates Management of Lesions to Exclude Melanoma.

This was a reliability cohort study conducted in a primary care skin cancer clinic in New South Wales, Australia. Data were collected between April 2019 and December 2019. This study assessed the reliability of a diagnostic classification system MOLEM for melanoma diagnosis. Nine certified pathologists comprised a panel whose goal was to give a histopathology report of histology slides based on the proposed MOLEM schema. (A diagram of flow of participants is found in Figure 1; pathologists’ characteristics and level of expertise are supplied in eTable 1 in the Supplement.) Images and data set are available for public access.^11^ Ethical approval was granted by the Bellberry Human Research Ethics Committee, and participants gave written informed consent.

### Participant Selection and Preparation of Histologic Cases

Post hoc power calculation and sample size calculation were not performed. Instead, we selected a pool of high-risk participants with lesions suspicious for melanoma that clinicians decided to excise to rule out melanoma. Suspicion of having melanoma was defined as an indication to perform testing, including signs and symptoms, and the presence of risk factors or results of previous medical tests. Baseline demographic information (Table 1) was collected from the participants such as age, sex, and previous medical and family history of melanoma. All participants underwent clinical evaluation, dermoscopy imaging, and subsequent excision biopsy of the suspicious lesion(s). Slides were prepared by conventional histologic method and stained with hematoxylin-eosin with immunohistochemical stains added if clinically indicated. A total of 198 patients were enrolled but the histopathology slides from 1 patient were not available; hence, 197 patients were included for histological analysis.

**Table 1.** Epidemiological Characteristics of Study Participants and Characteristics of Lesions

Characteristics	Patients, No. (%) (N = 197)
Age, mean (range), y	63.5 (23-92)
Sex	
Male	102 (51.8)
Female	95 (48.2)
History of melanoma	
Melanoma in situ	92 (46.7)
Invasive melanoma	51 (25.9)
**Lesions (n = 217)**	
Anatomic site/delphi location	
Trunk	116 (53.5)
Upper extremity	41 (18.9)
Lower extremity	30 (13.8)
Head and neck	27 (12.4)
Anogenital region	2 (0.9)
**Diagnoses (n = 1516)**	
MOLEM class^a^	
I	677 (44.7)
II	120 (7.9)
III	564 (37.2)
IV	114 (7.5)
V	55 (3.6)

Abbreviation: MOLEM, Management of Lesions to Exclude Melanoma.

^a^ MOLEM classes include the following: class I, benign naevus, mild atypia, seborrheic keratosis, lichenoid keratosis, cyst; class II, moderately atypical naevus, Spitz, deep penetrating naevus; class III, melanoma in situ, severely atypical naevus; class IV, invasive melanoma; and class V, basal cell carcinoma, squamous cell carcinoma in situ.

### Pathology Assessment

All lesions underwent conventional clinical diagnosis by the attending or referring physician prior to excision and histopathology assessment. The same slides were reviewed by up to 9 independent pathologists from Australia and/or the US for histopathologic diagnosis using the MOLEM schema. For logistical reasons, not all pathologists examined each lesion—a minimum of 5 pathologists made a diagnosis on the lesions. The 9 pathologists were presented with either digital histology slides or glass histology slides. Digital slide scanning was carried out with a pathology scanner (Leica Biosystems) at ×40 magnification, and then uploaded to a digital slide presentation platform (Pathpresenter). Glass histology slides and digital histology slides were considered equivalent for the purposes of this study. Pathologists were given access to standard patient data of each case, including patients’ age, sex, and basic clinical details present on the pathology request form. A total of 217 lesions were presented as glass or scanned slides. The principal investigator (I.K.) reviewed each case and chose 1 or more representative sections that included relevant immunohistochemistry stains.

Diagnoses were requested from each pathologist in a form of descriptive text and MOLEM assessment (Table 2). MOLEM schema consists of 5 classes. Class I lesions, which include benign naevus, naevus with mild atypia, seborrheic keratosis, lichenoid keratosis, and cyst, pose very low risk, and generally no further treatment is recommended. Class II lesions include moderately atypical naevus, Spitz, and deep penetrating naevus, which have low risk of progression and may merit narrow but complete excision (<5 mm margins). Class III lesions, such as melanoma in situ and severely atypical naevus, have higher risk of progression and may require complete excision (>5 mm but <10 mm margins). Class IV encompasses invasive melanoma and poses the highest risk of progression, and potentially warrants complete excision, with margins larger than 10 mm. Class V includes nonmelanoma skin cancer such as basal cell carcinoma and in situ squamous cell carcinoma, which requires definitive malignant-nonmelanoma management (eg, excision or other topical therapy). The suggested management for MOLEM classes I through IV was based on its diagnostic reference, MPATH-Dx. Pathologists were asked to assume the slides presented for each case were representative of the lesion and that the lesion went to the margins of the excision. Pathologists were also asked to score their diagnostic confidence from most (1) to least (5). The reference standard was defined by the most frequent MOLEM class level (ie, mode by majority diagnosis) of each case by all participating pathologists. In the case of a bimodal tie, the more severe diagnosis was chosen.

**Table 2.** MOLEM Reporting Schema for Classification of Skin Lesions

MOLEM class	Risk of progression	Suggested management	Examples
I	Very low risk	No further treatment or topical	Benign naevus, mildly atypical naevus, seborrhoeic keratosis, lichenoid keratosis, cyst
II	Low risk	Narrow but complete excision <5 mm	Moderately atypical naevus, Spitz, deep penetrating naevus
III	Higher risk	Complete excision with >5 mm but <10 mm margins	Melanoma in situ, severely atypical naevus
IV	Highest risk	Complete excision with >10 mm margins	Invasive melanoma
V	High risk	Definitive excision and malignant nonmelanoma management pathway	Basal cell carcinoma, squamous cell carcinoma in situ

Abbreviation: MOLEM, Management of Lesion to Exclude Melanoma.

### Statistical Analysis

Descriptive statistics were used to describe the characteristics of the sample and lesions. Interrater agreement (overall and by majority MOLEM class) and confidence ratings were assessed using Gwet AC1 coefficients^12^ with quadratic weighting applied because of the ordinal nature of the ratings for melanocytic and, separately, nonmelanocytic lesions.^12^ It must be noted that the MOLEM classes include both melanocytic and nonmelanocytic lesions, which presents a statistical challenge wherein the difference between nonmelanocytic classes I and V are a single step from each other. Therefore, lesions in class I were set as a value of “1” in the calculations and class V were given a value of “2”, whereas the other classes (II, III, and IV) were given their corresponding values vs class I. Class V lesions share suggested management courses with class II lesions, and so both had a set value of 2 for statistical analysis. The strength of agreement was classified as follows: agreement of less than 0.2 was poor, 0.21 to 0.40 was slight, 0.41 to 0.60 was fair, 0.61 to 0.80 was substantial, and 0.81 to 1.00 was almost perfect.^13^ Overdiagnosis was calculated by dividing the number of the number of diagnoses with MOLEM scores above the majority diagnosis by the total number of diagnoses. Underdiagnosis was calculated by dividing the number of diagnoses below the majority MOLEM score by the total number of diagnoses. Concordance analysis was also performed by calculating the number of cases that are concordant over the number of cases assessed. Excel (Microsoft) and Stata version 16.1 (StataCorp) were used for the analyses. A 2-sided *P* < .05 was considered significant.

## Results

One hundred ninety-seven patients were included in the study; the mean (SD) age of patients was 63.5 (15.8) years (range, 23-92 years), 102 (51.8%) were men, and 95 (48.2%) were women (Table 1). Overall, 217 index lesions were assessed with a total of 1516 histological diagnoses. Of 1516 diagnoses, 677 (44.7%) were classified as MOLEM class I; 120 (7.9%) as class II; 564 (37.2%) as class III; 114 (7.5%) as class IV; and 55 (3.6%) as class V. Class II interpretations showed 29 of 120 lesions being overcalled (ie, classed above the majority) and 30 of 120 lesions being undercalled (classed below the majority). Overall, compared with majority diagnosis, there was an overdiagnosis of melanoma of 5.3% (44 of 797 diagnoses), and 18.1% (123 of 678) of melanoma diagnoses were called benign.

### Concordance/Interrater Agreement

**Figure 2.**
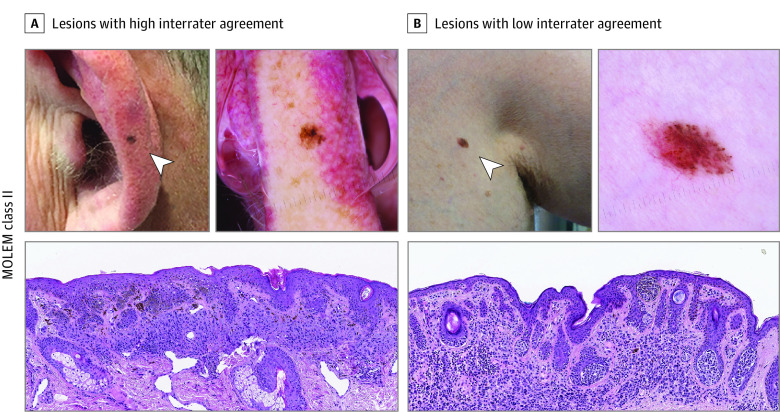
Example Lesions in MOLEM Class II MOLEM indicates Management of Lesions to Exclude Melanoma.

The mean (SD) number of raters (ie, pathologists) was 6.9 (1.1) for class I, 7.5 (1.2) for class II, 6.8 (1.1) for class III, 6.9 (1.4) for class IV, and 6.8 (0.9) for class V, respectively (Table 3). The concordance rate of MOLEM classes I through V were 88.6% (600 of 677 diagnoses), 50.8% (61 of 120 diagnoses), 76.2% (430 of 564 diagnoses), 77.2% (88 of 114 diagnoses), and 74.5% (41 of 55 diagnoses), respectively (eTable 3 in the Supplement). Figure 2 shows examples of lesions with high and low rates of concordance for MOLEM class II (examples for all classes are shown in eFigure 2 in the Supplement). The strength of interrater agreement by majority MOLEM class ranged from fair to almost perfect. Class II had the lowest interrater agreement (fair, 50.8%) with a quadratic weighted Gwet AC1 coefficient of 0.55 (95% CI, 0.44-0.66), while class V had the highest interrater agreement (almost perfect, 96.6%) with an AC1 coefficient of 0.94 (95% CI, 0.88-1.00) in both unweighted and weighted agreement analyses (Table 3). Classes III and IV had substantial interrater agreement (76.2% and 77.2%) with quadratic weighted Gwet AC1 coefficients of 0.82 (95% CI, 0.76-0.88) and 0.83 (95% CI, 0.67-0.89), respectively.

**Table 3.** Overall Pathologist Accuracy^a^

Agreement	Total lesions, No.	MOLEM class ratings				Confidence ratings			
		Total ratings, No.	Concordance with Majority MOLEM Class, %	Quadratic weighted interrater agreement		Total ratings, No.	Mean (SD) rating	Quadratic weighted interrater agreement	
				Agreement, %	Gwet AC (95% CI)			Agreement, %	Gwet AC (95% CI)
Pathologists’ ratings and majority MOLEM class	217	1530	79.7	94.7	0.86 (0.84-0.88)	NA	NA	NA	NA
Interrater agreement	217	1530	NA	91.3	0.76 (0.72-0.81)	1314	1.4 (0.7)	95.2	0.92 (0.90-0.94)
By majority MOLEM class^b^									
I	95	677	88.6	95.0	0.94 (0.91-0.96)	575	1.2 (0.3)	95.0	0.93 (0.90-0.96)
II	16	120	50.8	82.1	0.55 (0.44-0.66)	106	1.8 (0.7)	93.5	0.85 (0.79-0.91)
III	82	564	76.2	88.7	0.82 (0.76-0.88)	564	1.5 (0.7)	95.3	0.91 (0.88-0.93)
IV	16	114	77.2	89.0	0.83 (0.67-0.89)	114	1.3 (0.6)	96.5	0.94 (0.89-0.99)
V	8	55	74.5	96.6	0.94 (0.88-1.00)	55	1.1 (0.4)	97.5	0.97 (0.93-1.00)

Abbreviations: MOLEM, Management of Lesion to Exclude Melanoma; NA, not applicable.

^a^ The overall pathologist accuracy is shown compared with the majority diagnosis (ie, concordance rate); agreement between pathologist’s classification and the majority rating; and the overall interrater agreement (nonweighted and weighted). The strength of agreement was classified as follows: poor, <0.2; slight, 0.21-0.40; fair, 0.41-0.60; substantial, 0.61-0.80; almost perfect, 0.81-1.00. Concordance rate equaled the number of concordant case divided by the total number of cases assessed.

^b^ MOLEM classes include the following: class I, benign naevus, mild atypia, seborrheic keratosis, lichenoid keratosis, cyst; class II, moderately atypical naevus, Spitz, deep penetrating naevus; class III, melanoma in situ, severely atypical naevus; class IV, invasive melanoma; and class V, basal cell carcinoma, squamous cell carcinoma in situ.

The overall confidence level of each pathologist was rated as almost perfect, with an interrater agreement with Gwet AC1 coefficient of 0.92 (95% CI, 0.90-0.94). Overall, the reliability of MOLEM diagnosis confidence ratings was 95.2%, with a Gwet AC1 coefficient of 0.92 (95% CI, 0.90-0.94). Class II diagnosis confidence ratings showed the least mean (SD) values (1.8 [0.7]) of all classes with 93.5% agreement and a quadratic weighted Gwet AC1 coefficient of 0.85 (95% CI, 0.79-0.91) (eTable 2 in the Supplement). The highest diagnostic confidence ratings and agreement were found in class V diagnoses, which had mean confidence values of 1.1 (0.1) with a quadratic weighted Gwet AC1 coefficient of 0.97 (95% CI, 0.93-1.00).

### Variation in Diagnostic Labeling and Variation in Confidence vs Interrater Agreement

While some lesions were uniformly interpreted with similar diagnostic terms, other lesions showed substantial variability in diagnostic labeling (see eFigure 1 in the Supplement for histology images with different descriptions grouped in the same MOLEM class). This study involved pathologists from different parts of Australia and from other countries. It is quite likely that there are variations in terminologies and diagnoses. Moreover, there were variations in terms of diagnostic confidence and interrater agreement (see eFigure 2 and eFigure 3 in the Supplement for histology images with high diagnostic confidence but with low interrater agreement, or with low diagnostic confidence but with high interrater agreement).

## Discussion

The major finding of this study was that in a population of lesions excised to exclude melanoma and uniquely including melanocytic and nonmelanocytic lesions, the MOLEM schema was clinically useful. This finding is supported by the MOLEM interrater agreement alignment with previous studies and a high level of agreement was found within the pathologist confidence rating data. This study has shown effective lesion classification without prior knowledge or selection of lesion diagnosis. The data showed that dermatopathologists do not always agree upon a diagnosis, but statistical analysis reveals that dermatopathologists are aware of these difficult cases when diagnosing, which could be a point of entry for automated analysis in the future.

Additionally, the data from this study shows that, in an Australian population, there is an over- and underdiagnosis of melanoma (5.3% and 18.1%, respectively) compared with the estimated population-based statistics reported by Elmore et al^3^ using prevalence data from the US (8% overdiagnosed and 9% underdiagnosed). In this study and an associated report discussing only the nonmelanocytic lesions,^10^ we have proposed MOLEM as a modification to the MPATH-Dx diagnostic and management schema for classification of melanocytic lesions. Although the MPATH-Dx has been shown to be more concordant for the diagnosis of melanocytic lesions, the current study also reveals that there is variation in the terminology and over- and underdiagnosis of malignancy for nonmelanocytic lesions, similar to melanocytic lesions. Similar to Elmore et al,^3^ we have shown that most of the variability is in class II and class III lesions. A unique aspect of the current study was that the primary data set, including each benchmark diagnosis, was complete and made freely available, which adds value to this study by enabling future research.

Discordance in diagnosis is not unique to melanocytic lesions. Diagnoses made by physicians are not correct all the time. In fact, second reviews of images or specimens in radiology, pathology, and dermatology show potential for diagnostic error ranging from 10% to 50%.^14^ Disagreement between pathologists has also been reported for prostate, thyroid, and breast lesions.^3,4,7^

Another novel aspect of this study is the inclusion of confidence scores. There have been no studies, to our knowledge, that examine the association of confidence of pathological diagnosis with accuracy of diagnosis of lesions biopsied to exclude melanoma, or even difficult melanocytic lesions alone. Confidence in making diagnosis may sometimes influence accuracy of diagnosis. Ferrara et al^15^ suggested that more information available for melanocytic lesion improves pathologists’ confidence in diagnosis and leads to more accurate diagnosis. Clinically, for instance, in Benvenuto-Andrade et al^16^ the level of diagnostic confidence in clinical and dermoscopy examination of skin lesions that would be within MOLEM classes II and III (eg, dysplastic naevus and melanoma in situ) had lower confidence scores than lesions which would be classified as class I (eg, blue naevus, congenital naevus) and class IV (eg, malignant melanoma).^16^ In Meyer et al^8^ the physicians’ level of clinical diagnostic confidence was relatively insensitive to diagnostic accuracy and case difficulty. This contrasts with our outcome. We hypothesize that these similarities and differences are due to the unique nature of the morphological diagnosis of melanoma, which mainly relies primarily on pattern recognition. If confirmed, this hypothesis supports the combined use of automated image analysis with expert morphological assessment to achieve a higher level of diagnostic accuracy for melanoma. In the current study, pathologists showed the least confidence in the cases with the lowest levels of agreement.

In the nascent stages of AI in skin pathology, creating a data set that contains minimal bias in terms of disease classification and diagnosis remains a challenge as there are difficulties in deriving criterion standard diagnosis in pathology to be used in machine learning.^7^ Some inherent bias in the data used to train AI algorithm has been observed in the health care sphere.^17^ Interestingly, none of the recent papers looking at the AI diagnosis of images of skin lesions clinically and dermoscopically has mentioned the difficulty of developing a criterion standard to allow accurate histological diagnoses.^18^ As AI becomes more prevalent in assisting both clinical and histopathological diagnosis, developing a criterion standard for accurate histological diagnosis is vital. Using a diagnostic classification schema such as MOLEM can accommodate all lesions encountered when excluding melanoma. This makes the MOLEM schema viable for translation from the academic setting, where MPATH-Dx is commonly used, to the clinic. Translating MOLEM or a similarly scoped schema into practice will help address the issue of variation in diagnostic terminology; developing a viable clinical criterion standard will be beneficial for machine learning and, ultimately, patient outcomes.

There are a few potential methods to create this criterion standard. Such a standard could be defined in consensus meetings, similar to what was used in the development of the MPATH-Dx system.^4^ In addition, correlation with clinical and dermoscopic images has been shown to improve diagnostic certainty because in many cases, the clinical and dermoscopic impression can be more typical than the histological impression.^19,20,21^ Other methods to improve diagnostic certainty could include requesting second opinions including expert opinions, consensus discussions and diagnosis, additional sampling and slides, and ordering specialized tests.^5^ For example, Lohman et al^22^ described how newer immunohistochemical stains such as PRAME and p16 may be useful in helping to distinguish benign from malignant melanocytic lesions. Molecular diagnostics may also be an important source of objective diagnostic data.^23^

It is unclear which approach would be most beneficial when defining entities for training AI algorithms. However, oversimplifying classification systems might hinder the potential for machine-based learning algorithms to offer new, and previously unrecognized insights into disease biology. Adamson and Welch^7^ suggest that it may be useful to use information from a panel of pathologists and divide lesions into 3 categories: total agreement lesion is benign, total agreement the lesion is malignant, and disagreement whether the lesion is malignant. We believe using a classification schema such as MPATH-Dx or MOLEM that groups lesions with similar management strategies will help address the issue of variation in the diagnostic terminology, which will be beneficial for machine learning and developing a criterion standard.

### Limitations

This study had several limitations. First, the selection of the participant cohort was subject to selection bias. Second, the histologic classification of lesions was subject to observation bias, which was addressed by involving 9 independent pathologists masked to the histopathologic diagnosis.

Although the sample population of 217 lesions is comparable with previously published work in this area, the sample size is somewhat limiting.^24,25,26,27,28,29^ Additionally, the cases included in this study were a more clinically relevant spectrum of disease (eg, nonmelanocytic lesions) compared with previous studies^24,25,26,27,28,29^ that used curated data sets, which enabled large-scale diagnostic surveys and showed similar population-based results. One of the most interesting facts about this cohort was the higher number of previous melanomas in situ and invasive melanoma, which informed this cohort being considered as higher risk. Hence, a majority diagnosis was used as the comparator instead of the expert consensus diagnosis, and the diagnostic confidence score of each pathologist was included. This study was not limited by pathologists only having access to the clinically representative lesion because they also had access to multiple sections on 1 or more slides that included immunohistochemical stains, which was closer to the clinical pathological workflow used to diagnose a lesion. Moreover, pathologists involved had several years (ie, 3 to 29 years) of reporting melanocytic lesions in private pathology practices with referral populations from around Australia and in the US.

## Conclusions

In this study, the proposed schema for classifying lesions had good interrater agreement. A more clinically relevant concise diagnostic nomenclature and management hierarchy for lesions excised to exclude melanoma (MOLEM) including nonmelanocytic lesions has the potential to improve diagnostic agreement and reduce confusion over diagnosis compared with the previously reported MPATH-Dx schema. Moreover, numerical diagnostic schemes like MOLEM may be used as a natural bridge for AI-based diagnostic tool development.

## Notes

Supplement. **eTable 1.** Pathologist’s Characteristics and Level of Expertise **eTable 2.** Pathologist’s Confidence in Diagnosis **eTable 3.** Overall Pathologist Accuracy Compared to the Majority Diagnosis **eFigure 1.** Micrographic Images of H&E Slides (by Digital WSI) Showing Variations in Descriptive Labeling for melanocytic lesions **eFigure 2.** Dermoscopic and H&E Images (by Digital WSI) showing High and Low Inter-Rater Agreement **eFigure 3.** Dermoscopic Images (by Digital WSI) Showing Variations in Diagnostic Confidence and Inter-Rater Agreement

## References

[1] Corona R, Mele A, Amini M, . Interobserver variability on the histopathologic diagnosis of cutaneous melanoma and other pigmented skin lesions. J Clin Oncol. 1996;14(4):1218-1223. doi:10.1200/JCO.1996.14.4.1218 8648377

[2] Elder DE, Piepkorn MW, Barnhill RL, . Pathologist characteristics associated with accuracy and reproducibility of melanocytic skin lesion interpretation. J Am Acad Dermatol. 2018;79(1):52-59.e5. doi:10.1016/j.jaad.2018.02.070 29524584 PMC6016831

[3] Elmore JG, Longton GM, Carney PA, . Diagnostic concordance among pathologists interpreting breast biopsy specimens. JAMA. 2015;313(11):1122-1132. doi:10.1001/jama.2015.1405 25781441 PMC4516388

[4] Lott JP, Elmore JG, Zhao GA, ; International Melanoma Pathology Study Group. Evaluation of the Melanocytic Pathology Assessment Tool and Hierarchy for Diagnosis (MPATH-Dx) classification scheme for diagnosis of cutaneous melanocytic neoplasms: results from the International Melanoma Pathology Study Group. J Am Acad Dermatol. 2016;75(2):356-363. doi:10.1016/j.jaad.2016.04.052 27189823 PMC4958559

[5] Titus LJ, Reisch LM, Tosteson ANA, . Malpractice concerns, defensive medicine, and the histopathology diagnosis of melanocytic skin lesions. Am J Clin Pathol. 2018;150(4):338-345. doi:10.1093/ajcp/aqy057 30007278 PMC6116884

[6] Piepkorn MW, Barnhill RL, Elder DE, . The MPATH-Dx reporting schema for melanocytic proliferations and melanoma. J Am Acad Dermatol. 2014;70(1):131-141. doi:10.1016/j.jaad.2013.07.027 24176521 PMC3992990

[7] Adamson AS, Welch HG. Machine learning and the cancer-diagnosis problem—no gold standard. N Engl J Med. 2019;381(24):2285-2287. doi:10.1056/NEJMp1907407 31826337

[8] Meyer AN, Payne VL, Meeks DW, Rao R, Singh H. Physicians’ diagnostic accuracy, confidence, and resource requests: a vignette study. JAMA Intern Med. 2013;173(21):1952-1958. doi:10.1001/jamainternmed.2013.10081 23979070

[9] Ackerman AB. Differential Diagnosis in Dermatopathology II. 2nd ed. American Journal of Dermatopathology; 1989.

[10] Katz I, Azzi A, Lilleyman A, . MOLEM (Management of Lesions Excised to Exclude Melanoma): variability in the histopathological diagnosis of non-melanocytic lesions excised to exclude melanoma. Dermatol Pract Concept. Accepted manuscript. 2021. doi:10.5826/dpc.1104a94 PMC8648420 35024222

[11] Katz I, O’Brien B, Clark S, . The management of lesions to exclude melanoma (MOLEM). University of South Australia research data access portal. Published November 10, 2021. Accessed November 17, 2021. https://data.unisa.edu.au/dap/Dataset.aspx?DatasetID=714038

[12] Gwet KL. Computing inter-rater reliability and its variance in the presence of high agreement. Br J Math Stat Psychol. 2008;61(Pt 1):29-48. doi:10.1348/000711006X126600 18482474

[13] Landis JR, Koch GG. The measurement of observer agreement for categorical data. Biometrics. 1977;33(1):159-174. doi:10.2307/2529310 843571

[14] Graber ML. The incidence of diagnostic error in medicine. BMJ Qual Saf. 2013;22(suppl 2):ii21-ii27. doi:10.1136/bmjqs-2012-001615 23771902 PMC3786666

[15] Ferrara G, Argenyi Z, Argenziano G, . The influence of clinical information in the histopathologic diagnosis of melanocytic skin neoplasms. PLoS One. 2009;4(4):e5375. doi:10.1371/journal.pone.0005375 19404399 PMC2671836

[16] Benvenuto-Andrade C, Dusza SW, Hay JL, . Level of confidence in diagnosis: clinical examination versus dermoscopy examination. Dermatol Surg. 2006;32(5):738-744. doi:10.1097/00042728-200605000-00034 16706773

[17] Richens JG, Lee CM, Johri S. Improving the accuracy of medical diagnosis with causal machine learning. Nat Commun. 2020;11(1):3923. doi:10.1038/s41467-020-17419-7 32782264 PMC7419549

[18] Esteva A, Kuprel B, Novoa RA, . Dermatologist-level classification of skin cancer with deep neural networks. Nature. 2017;542(7639):115-118. doi:10.1038/nature21056 28117445 PMC8382232

[19] Argenziano G, Soyer HP. Dermoscopy of pigmented skin lesions—a valuable tool for early diagnosis of melanoma. Lancet Oncol. 2001;2(7):443-449. doi:10.1016/S1470-2045(00)00422-8 11905739

[20] Curchin C, Wurm E, Jagirdar K, Sturm R, Soyer P. Dermoscopy, reflectance confocal microscopy and histopathology of an amelanotic melanoma from an individual heterozygous for MC1R and tyrosinase variant alleles. Australas J Dermatol. 2012;53(4):291-294. doi:10.1111/j.1440-0960.2012.00882.x 22497519

[21] Menzies SW, Moloney FJ, Byth K, . Dermoscopic evaluation of nodular melanoma. JAMA Dermatol. 2013;149(6):699-709. doi:10.1001/jamadermatol.2013.2466 23553375

[22] Lohman ME, Steen AJ, Grekin RC, North JP. The utility of PRAME staining in identifying malignant transformation of melanocytic nevi. J Cutan Pathol. 2021;48(7):856-862. doi:10.1111/cup.13958 33433032

[23] Clarke EL, Munnings C, Williams B, Brettle D, Treanor D. Display evaluation for primary diagnosis using digital pathology. *J Med Imaging (Bellingham)*. 2020;7(2):027501. 32341938 10.1117/1.JMI.7.2.027501 PMC7177184

[24] Elmore JG, Barnhill RL, Elder DE, . Pathologists' diagnosis of invasive melanoma and melanocytic proliferations: observer accuracy and reproducibility study. BMJ. 2017;357. doi:10.1136/bmj.j2813 PMC5485913 28659278

[25] Farmer ER, Gonin R, Hanna MP. Discordance in the histopathologic diagnosis of melanoma and melanocytic nevi between expert pathologists. Hum Pathol. 1996;27(6):528-531. doi:10.1016/S0046-8177(96)90157-4 8666360

[26] Hawryluk EB, Sober AJ, Piris A, . Histologically challenging melanocytic tumors referred to a tertiary care pigmented lesion clinic. J Am Acad Dermatol. 2012;67(4):727-735. doi:10.1016/j.jaad.2012.02.036 22521204

[27] Onega T, Barnhill RL, Piepkorn MW, . Accuracy of digital pathologic analysis vs traditional microscopy in the interpretation of melanocytic lesions. JAMA Dermatol. 2018;154(10):1159-1166. doi:10.1001/jamadermatol.2018.2388 30140929 PMC6233746

[28] Shoo BA, Sagebiel RW, Kashani-Sabet M. Discordance in the histopathologic diagnosis of melanoma at a melanoma referral center. J Am Acad Dermatol. 2010;62(5):751-756. doi:10.1016/j.jaad.2009.09.043 20303612

[29] van Dijk MC, Aben KK, van Hees F, . Expert review remains important in the histopathological diagnosis of cutaneous melanocytic lesions. Histopathology. 2008;52(2):139-146. doi:10.1111/j.1365-2559.2007.02928.x 18184263

