# Supplementary material for: Assessment of a Diagnostic Classification System for Management of Lesions to Exclude Melanoma
Source: JAMA Netw Open. 2021 Dec 10;4(12):e2134614. doi: 10.1001/jamanetworkopen.2021.34614 (PMC8665368; doi:10.1001/jamanetworkopen.2021.34614)
Supplement: Supplement. — eTable 1. Pathologist’s Characteristics and Level of Expertise eTable 2. Pathologist’s Confidence in Diagnosis eTable 3. Overall Pathologist Accuracy Compared to the Majority Diagnosis eFigure 1. Micrographic Images of H&E Slides (by Digital WSI) Showing Variations in Descriptive Labeling for melanocytic lesions eFigure 2. Dermoscopic and H&E Images (by Digital WSI) showing High and Low Inter-Rater Agreement eFigure 3. Dermoscopic Images (by Digital WSI) Showing Variations in Diagnostic Confidence and Inter-Rater Agreement [file jamanetwopen-e2134614-s001.pdf]

Supplemental Online Content

Katz I, O'Brien B, Clark S, et al. Assessment of a diagnostic classification system for management of lesions to exclude melanoma. *JAMA Netw Open.* 2021;4(12):e2134614.
doi:10.1001/jamanetworkopen.2021.34614

eTable 1. Pathologist's Characteristics and Level of Expertise

eTable 2. Pathologist's Confidence in Diagnosis

eTable 3. Overall Pathologist Accuracy Compared to the Majority Diagnosis

eFigure 1. Micrographic Images of H&E Slides (by Digital WSI) Showing Variations in Descriptive Labeling for Melanocytic Lesions

eFigure 2. Dermoscopic and H&E Images (by Digital WSI) Showing High and Low Inter-Rater Agreement.

eFigure 3. Dermoscopic Images (by Digital WSI) Showing Variations in Diagnostic Confidence and Inter-Rater Agreement

This supplemental material has been provided by the authors to give readers additional information about their work.

© 2021 Katz I et al. *JAMA Network Open.*

Pathologists' Characteristics and Level of Expertise

Pathologist's demographic	Qualification	Setting of practice	Referral population	Number of years reporting melanocytic lesion	Proportion of melanocytic lesions within overall caseload	Provide 2nd opinion for melanocytic lesions	Terminology used / avoided
I.K.,	MBChB; BSc (Med)(Hons); FRCPA	Private pathology	Australia	25	Approximately 20%	Yes, predominantly within practice	Avoid tern dysplastic nevus, grade atypia but avoid using severe atypia, do not use MPATH-Dx, Avoid MELTUMP
B.O.B.	MBBS (Hons 1st class); FRCPA; Cert Dermatoscopy	Private Pathology	Queensland, Northern NSW, Australia	9 years	Approximately 20%	Yes, within practice and for regional private laboratories	Commonly used term 'Dysplastic naevus'<br><br>Commonly grade level of atypia (e.g. moderate, severe)<br><br>Do not use MPATH-Dx
S.C.	MBChB; FRCPA	Private<br>Pathology	Australia	25	20%	Yes	Avoid dysplastic naevus, avoid grading, favour benign or malignant or uncertain
C.T.	BS; MD; FAAD	Private Pathology	USA	25	30%	Within practice and provide 2nd opinions to outside clinicians and pathologists	Avoid grading dysplastic. Generally, use "atypical" or not.
B.S.,	BSc (Med) (Hons). MBChB (Hons)	Private pathology	USA	29	Approximately 30 %	Yes, within practice and regional to clinicians	Do not use MPATH-Dx<br>Grade dysplastic depending on which clinician
L.L.	MBBS, MD, PhD, FRCPA	Private pathology	Australia – community	3	Approx. 20%	No	Avoid term dysplastic

© 2021 Katz I et al. *JAMA Network Open.*

			Pathology Practice				naevus, do not use MPATH-Dx
M.G.		Private pathology	Australia community Pathology Practice	3 year	Approx. 20%	No	Avoid term dysplastic naevus, do not use MPATH
R.P.	MBBS (UQ) MPhil (Dermatopathology) (UQ)<br>Dip Practical Dermatology (UW)<br>FRCPA<br>FRACGP<br>Dip SCCA (Dermoscopy)	Private pathology	Australia community Pathology Practice	14 years	Approximately 20%	Yes, predominantly within practice.<br>Regularly use national public experts	Avoid MELTUMP<br>Two-tier system of grading atypia
R.S.	MBBS (UQ) MPhil (Dermatopathology) (UQ)<br>Dip Practical Dermatology (UW)<br>FRCPA<br>FRACGP<br>Dip SCCA (Dermoscopy)	Private pathology	Australia community Pathology Practice	14 years	Approximately 20%	Yes, predominantly within practice Regularly use national public experts	Avoid MELTUMP<br>Two-tier system of grading atypia
T.Y.	MBBS FRCPA	Private pathology	Australia community	8 years	Approx. 20%	Yes, predominantly within practice	Avoid "dysplastic", grade dysplasia, do not use severe, do not use MPATH-Dx
V.S.	MBBS, FRCPA	Private pathology	Australia community	6years	10%	Yes, predominantly within practice	Do not use term dysplastic naevus, do not use MPATH-Dx

eTable 1. Pathologist's characteristics and level of expertise.

© 2021 Katz I et al. *JAMA Network Open.*

**Consensus MOLEM diagnosis**	**Number of diagnoses**	**Mean score ± SD**	**Range (Min to Max)**
**Class I**	677	1.3 ± 0.3	1 to 5
**Class II**	120	1.8 ± 0.2	1 to 4
**Class III**	564	1.5 ± 0.4	1 to 5
**Class IV**	114	1.3 ± 0.3	1 to 4
**Class V**	41	1.1 ± 0.1	1 to 3
**Total**	1516	1.4 ± 0.4	1 to 5

eTable 2. Pathologist's confidence in diagnosis

© 2021 Katz I et al. *JAMA Network Open.*

**Majority MOLEM diagnosis**	**Pathologists' interpretation**					**Total diagnoses**	**Concordance (%)**
	**Class I**	**Class II**	**Class III**	**Class IV**	**Class V**		
**Class I**	**600**	47	15	0	15	677	88.6%
**Class II**	30	**61**	22	7	0	120	50.8%
**Class III**	62	49	**430**	23	0	564	76.2%
**Class IV**	7	5	14	**88**	0	114	77.2%
**Class V**	4	0	0	0	**37**	41	90.2%
**Total**	703	162	481	118	52	1516	

eTable 3. Overall pathologist accuracy compared to the majority diagnosis.

© 2021 Katz I et al. *JAMA Network Open.*

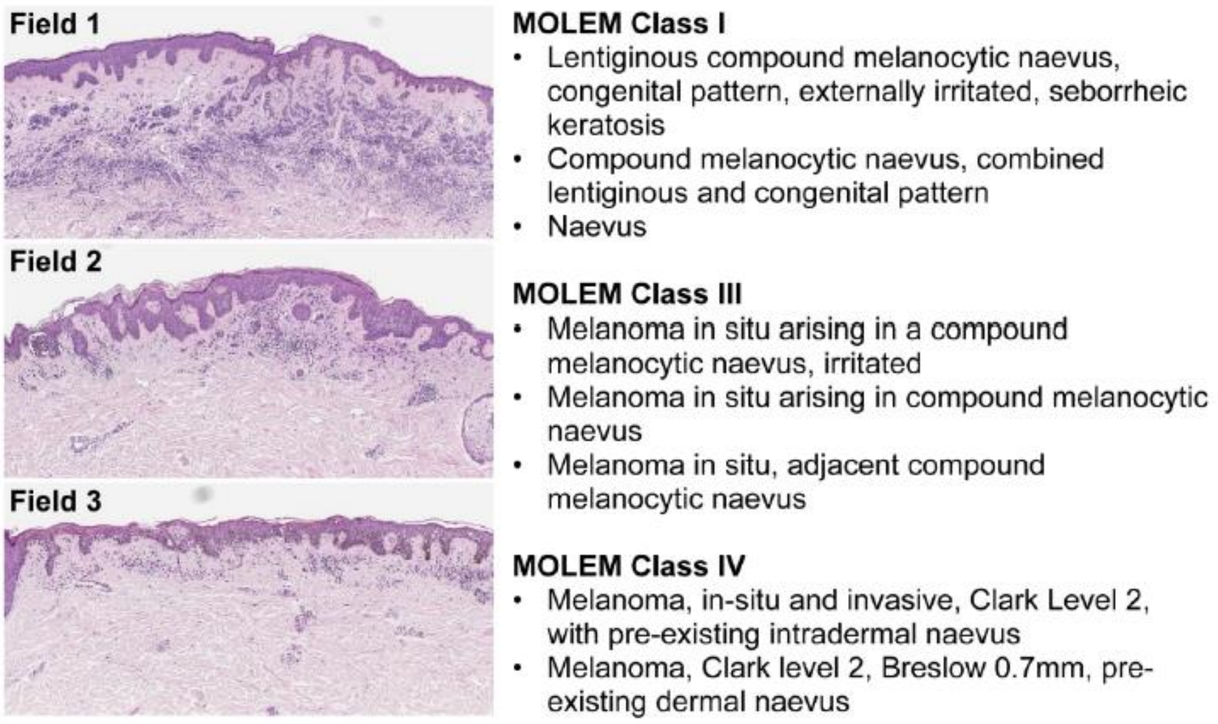

eFigure 1. Micrographic Images of H&E Slides (by digital WSI) Showing Variations in Descriptive Labeling for melanocytic lesions. The left panel shows three representative micrographic images from the digital whole slide image (WSI) of the H&E slide (magnification approximately 100x) from a 42-year-old female from the back (lesion 17) while the right panel shows the wide variation in the terminology used for this case and the different classes assigned to the lesion by eight pathologists

© 2021 Katz I et al. *JAMA Network Open.*

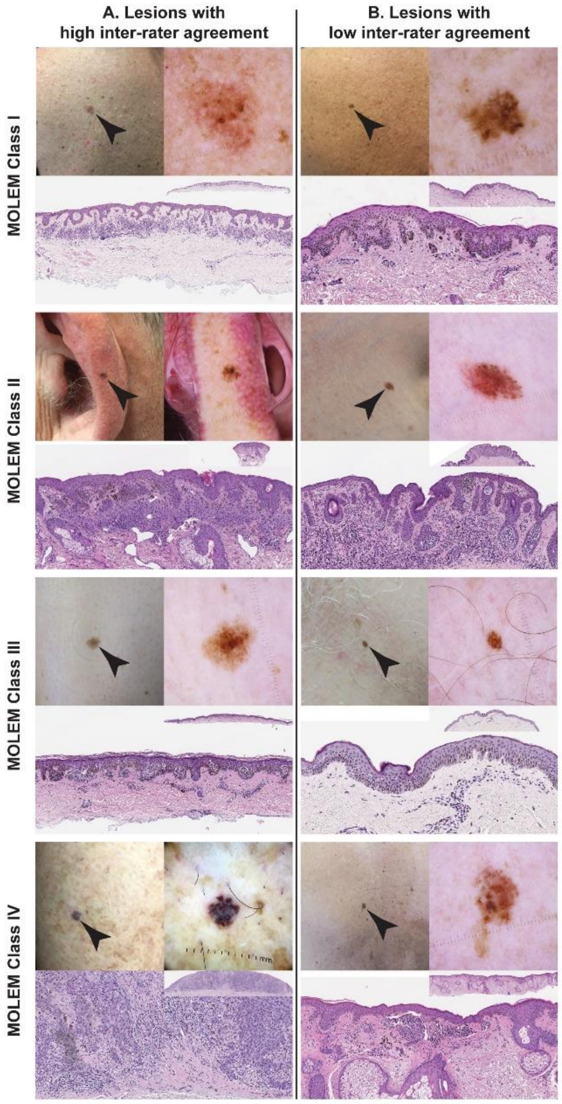

eFigure 2. Dermoscopic and H&E Images (by digital WSI) showing High and Low Inter-Rater Agreement. The clinical, dermoscopic and representative photomicrograph from the digital WSI of the H&E slide from four lesions showing highest levels of agreement. MOLEM class I, high agreement. A lesion 4 from the back of a 59-year-old female. Clinically and dermoscopically the lesion shows a broad, raised melanocytic lesion on sun-damaged skin. Dermoscopy shows uniform pigment network with some central thickening and increased vascularity. The WSI show a compound melanocytic naevus which all pathologists placed in class I. MOLEM class II, high agreement. A lesion 52a from the lesion from the ear of a 52-year-old male. Clinically and dermoscopically the lesion shows a small, pigmented macule with irregular pigmented network with grey follicular openings and black globules on dermoscopy. Six of nine pathologists graded this a class II and included diagnoses such as moderately atypical naevus, naevus of special site and spitzoid naevus. MOLEM class III, high agreement. A lesion 39 from the shin of a 46-year-old female. Clinically and dermoscopically the lesion shows a solitary uniformly pigmented macule on pale skin with homogenous peripheral pigment with central thickened network and follicular opening, extending to12 o'clock with brown dots and globules. The WSI shows MIS (class III) which all pathologists agreed upon. MOLEM class IV, high agreement. A lesion 44 from the leg of a 78-year-old female. Clinically the lesion shows a raised dark pigmented macule on sun-damaged skin. On dermoscopy the lesion consists of brown and black globules, blue-white veil and no obvious network. The WSI show an invasive melanoma which all pathologists graded as class IV. MOLEM class I, low agreement. A lesion 6 from the back of a 70-year-old male. Clinically and dermoscopically the lesion shows a raised, irregular pigmented macule on sun-damaged skin with asymmetry of pigment with loss of network, black dots and early pseudopods at 1 o'clock. The WSI show a naevus which pathologists classified from I to III (totally banal naevus to severe atypia). MOLEM class II, low agreement. A lesion 32 from the lesion from the chest of a 62-year-old male. Clinically and dermoscopically the lesion shows a raised pigmented macule with brown globules, inverse network and

© 2021 Katz I et al. *JAMA Network Open.*

increased vascularity. The pathologists graded this from class I to class IV with diagnoses ranging from naevus to invasive melanoma. MOLEM class III, low agreement. A lesion 135 from the chest of a 53-year-old male. Clinically and dermoscopically the lesion shows a small, pigmented macule with uniform pigment and thickened network. The pathologists graded this from class I to class III with diagnoses ranging from naevus to MIS. MOLEM class IV, low agreement. A lesion 152b from the back of a 65-year-old male. Clinically the lesion shows an irregular pigmented macule on sun-damaged skin. Dermoscopy shows asymmetry with features of regression with inverse network and grey dots. The pathologists graded this from class I to class IV with diagnoses ranging from naevus to invasive melanoma.

© 2021 Katz I et al. *JAMA Network Open.*

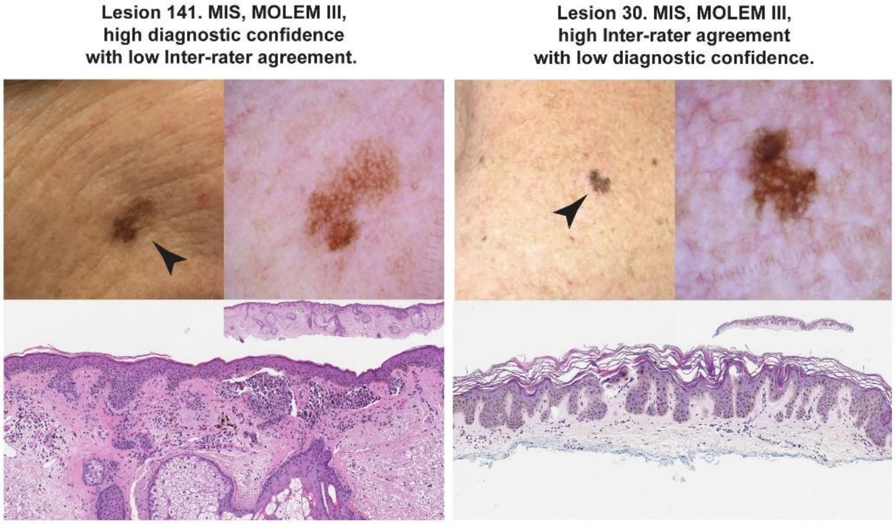

eFigure 3. Dermoscopic Images (by digital WSI) Showing Variations in Diagnostic Confidence and Inter-Rater Agreement. The left panel is lesion 141 from the forehead of a 58-year-old female. Clinically and dermoscopically the lesion shows a broad pigmented macule on sun-exposed skin with asymmetrical brown dots and globules. The pathologists were split about whether this was MIS or invasive melanoma (3/6 and 2/6, respectively) but were quite confident (1 for all 6) in their diagnosis. The right panel is lesion 30 from the shoulder of a 70-year-old female. Clinically the lesion shows an asymmetrical flat macule on sun-damaged skin. Dermoscopy shows irregular asymmetrical network with scattered black and grey dots. Most of the pathologists called this a class III MIS (7/8), but the degree of confidence was lower (2.14 ± 0.90, mean ± standard deviation).

© 2021 Katz I et al. *JAMA Network Open.*
